# Effects of a multi-level intervention on hookah smoking frequency and duration among Iranian adolescents and adults: an application of socio-ecological model

**DOI:** 10.1186/s12889-021-10219-8

**Published:** 2021-01-21

**Authors:** Fatemeh Bakhtari Aghdam, Nader Alizadeh, Haidar Nadrian, Christoph Augner, Asghar Mohammadpoorasl

**Affiliations:** 1 Road Traffic Injury Research Center, Tabriz University of Medical Sciences, Tabriz, Iran; 2 Department of Health Education & Promotion, Tabriz University of Medical Sciences, Tabriz, Iran; 3 Institute for Human Resources Research in Health Care, University Clinics of the Paracelsus Medical University, Salzburg, Austria; 4 Department of Biostatistics and Epidemiology & Health and Environment Research Center, Tabriz University of Medical Sciences, Tabriz, Iran

**Keywords:** Hookah, Tobacco smoking, Socio-ecological model, Multi-level intervention

## Abstract

**Background:**

The present study aimed to investigate the effects of a multi-level intervention on hookah smoking frequency and duration among Iranian adolescents and adults.

**Methods:**

In this study, two comparable cities in Iran were selected to participate in an intervention program based on a social-ecological model (SEM). In each city, 133 hookah smokers in coffee houses were selected. Environmental changes in coffee houses such as serving light foods and games were conducted. A virtual group named “no hookah” was established on the Telegram application to train participants in the intervention group. Messages, pictures, and short videos were sent to the participants through that virtual network. The frequency and duration of hookah consumption were assessed in both groups at baseline and after the intervention.

**Results:**

The frequency of hookah consumption decreased in 72.6% of participants in the intervention group (vs. 6.3% in the control group), and the duration of hookah consumption per session decreased in 39.5% of participants in the intervention group (vs. 5.5% in the control group).

**Conclusions:**

Using multi-level interventions through a social-ecological model can reduce hookah consumption in adults.

**Supplementary Information:**

The online version contains supplementary material available at 10.1186/s12889-021-10219-8.

## Background

Several studies show that the harmful effects of smoking hookahs are even more serious than smoking cigarettes. Research indicates that smoking hookah can result in health problems such as cancers, and cardiovascular and chronic respiratory diseases [1, 2].

The prevalence of hookah smoking across Iran has been reported to be between 3.5% [3–5]and 44.3% [6]. In many in the Eastern Mediterranean Region (EMRO)countries, smoking hookah has reached 20 to 30% among adults and its consumption rate among younger individuals is increasing [7, 8].

As far as we know, the evaluation of interventions carried out for reducing hookah smoking includes a study conducted by Huang et al. on the peers’ influence and the effects of online and offline networks of friends on using narcotics [9], a study by Haddad on increasing knowledge through a web-based method [10] and Lipkus et al’s study on the effect of receiving information on the perception of risk and increase in concern about the harmful effects of smoking hookah [11], as well as educational behavioral interventions and bupropion use in the countries of Egypt, Pakistan and the United States [11–13]. In all the studies mentioned above, more intervention studies are suggested. Recent researches have focused more on individual behavior and has neglected environmental and social variables. However, recent evidence from Iran shows that environmental and social factors might affect an individual’s behavior and their choice to smoke hookahs. Such factors include having a friend who smokes a hookah, low social support for the individual [5, 14], an environment that encourages people to smoke hookahs, lack of healthy entertainment for the youth that can be an alternative to drug consumption, the common use of hookahs among families and groups of friends and it’s being a norm in the public culture [15]. Also, due to the increase in smoking hookahs, it seems necessary to apply appropriate intervention to reduce hookah consumption [16]. In the field of public health, ecological models describe people’s interactions with their physical and socio-cultural surroundings [17]. The socio-ecological model (SEM) examines the health issue and its determinants with a multilevel approach, from individual and interpersonal levels to organizational and political levels. In Iran, the SEM frameworks have widely been used to treat different health problems [17, 18].

Since the smoking of hookahs has increased among the Iranian youth [5, 15] and because a behavior such as smoking hookahs should be considered in a social context, the best way to reduce the spread of using hookahs in society appears to be the application of widespread social programs as well as the formation of multiple channels of control [19].

Up to now, no multi-level interventions have been conducted using SEM analysis. Therefore, the present study was conducted to investigate (1) the conditions of smoking hookahs and (2) to design, apply, and evaluate a low cost and multi-faceted intervention program based on an SEM to reduce hookah smoking among adolescents and adults.

## Methods

This study was a quasi-experimental field study with a control group design conducted in the coffee shops of Hashtrud (intervention group) and Qarah-Aghaj (control group) counties in Eastern Azerbaijan province, Iran. The study was conducted in two phases. In the first phase (pre-test), the frequency and the determinants of hookah smoking behavior were investigated based on the Socio-ecological model (SEM) to find the significant predictors of the behavior among the participants. Based on the results found in the pre-test phase, a health promotion intervention program was designed and implemented in Hashtrud County, as the setting of intervention. The underlying idea is that interventions should include multilevel strategies focusing on individual behavior, social and environmental levels [20]. The following aspects were emphasized during the intervention: individual (education based on telegram), social approaches (create a social network, social support of peers and reward to decrease hookah use), and physical environmental levels (changes in coffees for example serving desserts and different kinds of drinks and healthy foods also were provided with intellectual game accessories).

### Instrumentation

The instruments used in the present study included a demographic data form, three items about the frequency of hookah smoking, hookah use-related individual and social level factors questionnaires, and an environmental level factors checklist. The questionnaires applied to assess the individual-level factors and perceived rewards(from the social level factors)were developed and validated in a previous study [21].

To assess the validity of the researcher-made instruments, a panel of 10 scholars (in the fields of health education and behavior, sociology, psychology, and epidemiology) reviewed and assessed the items, orally, and evaluated the appropriateness and relevance of the items to the participants. The scholars were all faculty members with field experience in tobacco use research and practice. They confirmed the items to be representative of the constructs to confirm the content validity of the instruments. We used the experts’ feedback on the instruments to revise and modify the items. Content Validity Ratio (CVR) with the feature of “necessity” and Content Validity Index (CVI) with two features of “relevance” and “sufficiency of each construct” were measured. To examine the utility of the scales and to identify the problems/benefits associated with the design, the instruments were pilot-tested by a sample of 30 hookah users not included in the final study. We used the data to estimate the internal consistency of the scales using Cronbach’s Alpha coefficient. Following consultation with the multidisciplinary team, the first draft was prepared.

The demographic data form included age, level of education, marital status, employment status, and level of income.

The frequency of smoking was investigated by applying three researcher-made self-report items. The items were as follow: “In the previous 3 months, how many times per week have you smoked hookah?”, “How long is the duration of your hookah smoking per serving?” and “How many cigarettes have you smoked in the previous seven days?” The hookah use questionnaire is included as the additional file 1. The CVI and CVR scores for the items of the frequency of smoking were 91.0 and 86.6, respectively.

The scales of individual-level factors included perceived sensitivity (5 items) and perceived severity (8 items). Examples of the items for perceived susceptibility and severity were “Using hookah will increase my chance for getting lung cancer “, and “Smoking hookah will reduce my chance for getting a suitable job”, respectively. Perceived internal and external rewards acquired from hookah use were also investigated applying a nine-item scale. One item, as an example, was “By smoking hookah, I feel that I am grown-up and feel like a man”. The triple scales were rated based on a five-point Likert-type scaling (ranging from 1 = totally disagree to 5 = totally agree). The scores of the scales were then summed to acquire a total score. The maximum total score for perceived susceptibility, severity, and rewards were 25, 40, and 45, respectively. The higher the scores in the triple scales indicated the higher levels of perceived susceptibility, severity, and internal and external rewards among the individuals to smoke a hookah. All scales are developed and validated in a previous study in Iran [21, 22].

Perceived social support questionnaire including 14 items was applied to assess the social level factors. Examples of the subjects presented in the items included “encouraged you not to go to the coffee shops to smoke hookah” and “encouraged you not to smoke hookah”. The response format was based on a 5-point Likert-type scale (from never [1] to always [5]). The total score was ranged from 14 to 70. The higher the score, the higher the level of social support was perceived by the individuals. Social support for hookah smoking questionnaire is included as the additional file 2. CVI and CVR values for the scale were 90.64 and 74.42, respectively, and the alpha Cronbach’s coefficient was 0.72.

A researcher-made 11-item environmental checklist was used to assess the environmental level factors. A dichotomous Yes/No scale was considered as a response format. An example of the items in the checklist was “Is there any game/ entertainment tool available in the coffee shop?” The environmental checklist for coffee shops is included as the additional file 3.

### Sampling and data collection

To conduct the study, Hashtrud County was considered as the setting to conduct the intervention. All coffee shops in Hashtrud (11 cases as intervention group) and Qarah-Aghaj (center of Charuymaq County) (7 cases as the control group) were selected as the study centers. We did not randomize the intervention and control groups between the coffee shops in Hashtrud and Qarah-Aghaj, because in these two small cities where the study was done, people know each other and have high interaction. So, if we randomized the intervention and control groups between the coffee shops in the cities, there would be a high possibility of contamination bias.

**Fig. 1.**
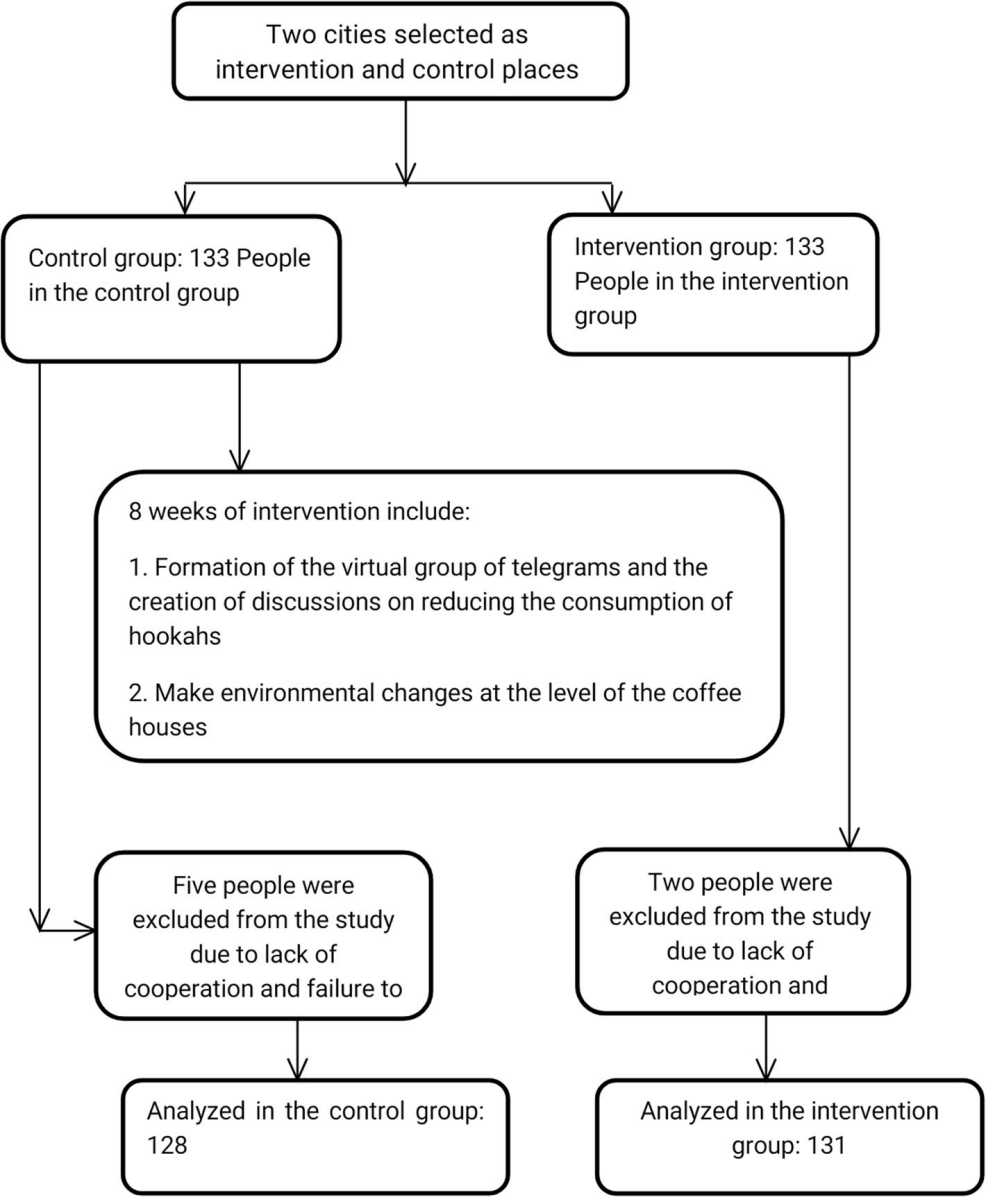
Chart: Hookah use reduction procedure in a flowchart (Iran, 2017)

As primary coordination, the administrators in the coffee shops were explained about the purpose of the study. All the coffee shop owners accepted to cooperate with the team of research, except for one, who was removed from the list of participating coffee shops. Then, the purpose of the study was explained to the hookah smokers in the coffee shops and they were invited to participate in the study. Informed consent was obtained from both the administrators and the hookah smokers who accepted to participate in the study and all signed consent forms. They were also assured of the confidentiality of their information. Along with data collection, applying the SEM-based questionnaire, the environmental checklist was also filled out based on the observations conducted by the second author. In each County, 133 hookah smokers at the coffee shops were included in the study. The flowchart of the study is illustrated in Fig. 1.

The intervention was performed in all Hashtrud coffee shops for 8 weeks from October to December 2017. Those who were older than 15 and younger than 35 years of age, had the willingness to quit hookah smoking in the following 6 months, were a daily user of the Telegram virtual network, and smoked hookah in the previous 12 months were included in the study.

Exclusion criteria were using drugs and narcotics other than cigarettes and the tobacco used in hookah, illiteracy, not having a smartphone, having plans to quit hookah smoking, and suffering from psychological disorders or mental problems such as depression.

### Interventional program

Based on the pre-test data analyses, the problems and weak points at the individual, environmental, and social level factors related to hookah smoking were identified. Therefore, the interventional program was designed and implemented as follows: A virtual group named “No to hookah smoking” was established on the Telegram application to train the participants in the intervention group, informally. Telegram application is a virtual network such as skype. Most Iranian people get used to Telegram as a social network [23].. Successful interventions had been performed using the Telegram network in Iran [24].

To promote their level of perceived sensitivity and severity, the participants were encouraged to share the group members with text messages, pictures and short videos regarding the outcomes of hookah smoking, health disadvantages of the behavior, the mechanisms via hookah smoking may damage human health, the ways, and benefits of quitting hookah use and how to resolve the obstacles, how to alleviate and control the temptation to smoke hookah, and alternatives for hookah smoking in the coffee shops. We initiated a group discussion to talk about the contents. To promote internal and external rewards among the participants, potential alternative rewards that may be achieved by reducing or quitting hookah use were discussed among the group members. The group members were socially supported by the managers of the group and their peers. The members received a variety of informational, emotional, and instrumental support. Group members rewarded each other with verbal encouragement as well as low internet service charges.

The resources and costs needed to implement the program included the coffee shops, the cost of internet service provision, and the time required to send messages. The key strategies applied to design the program were network-based education, consciousness-raising, environmental reconstruction, environmental reevaluation, role modeling, and perceived threat rising. The main processes applied to implement the program were experimental informal teaching methods (incorporating in the virtual social network and discussing the messages in the virtual group and reaching census, problem-solving discussions), discussing the early negative consequences of hookah smoking (e.g., coughs early in the mornings), and revisiting the services provided to customers in the coffee shops.

The training team at the group consisted of a public health expert and two researchers of the study (an epidemiologist and a health educationist) who managed the virtual group with 614 members. Also, 11 individuals, who used to smoke hookah but have then managed to quit, were included in the group, as laypersons. They played the role of peers in the virtual group and discussed different ways of quitting hookah use with the group members.

To make supportive environmental changes, some new services were provided to customers in the coffee shops. To help the participants in accessing healthy food choices in the coffee shops, the administrators were requested to have serving desserts and different kinds of drinks and healthy foods like lentil soup, omelet, ice-cream, figs, and broad beans. The participants in the coffee shops were also provided with intellectual game accessories (like chess, mensch, and others) as alternative entertainment instruments. Moreover, hookah smoking-related posters and banners with specific focuses were installed close to the coffee shops. Summary of interventions in different levels of SEM showed in Table 1.

**Table 1:** Intervention in different levels of the socio-ecological model

Levels of the socio-ecological model	Intervention
Individual-level	A virtual group was established on the Telegram application. Text messages, pictures, and short videos are shared in the Telegram group to promote perceived sensitivity, severity, benefits of quitting hookah use, and resolution of quitting barriers.
Social environment	Group members rewarded each other with verbal encouragements, low internet service charges, informal, emotional, and instrumental support by the managers of the group and the peers, and give internal and external rewards for who reducing or quitting hookah use.
Physical environment	The administrators were requested to have serving desserts and different kinds of drinks and healthy foods like lentil soup, omelet, ice-cream, figs, and broad beans, also were provided with intellectual game accessories (like chess, mensch, and others) as alternative entertainment instruments in coffee shops. Hookah smoking-related posters and banners were installed close to the coffee shops.

Two weeks after completing the implementation of the program, the questionnaires were again completed by the participants in the experimental and control groups.

The protocol of the study was approved by the Ethics Committee in an Iranian medical sciences university [Ethics code: IR.TBZMED.REC.1396.175].

### Data analysis

SPSS version 23 was used to analyze the data. Descriptive statistics were calculated to determine mean and standard deviation in quantitative data and frequency and percentage in qualitative data. Independent t-test and Chi-square were used to compare the mean between the groups. Logistic models were used to evaluate the effect of the multidimensional intervention on changes in the frequency and duration of hookah consumption by controlling for potential confounders. We used analysis of covariance test to compare the means after adjustment for the dependent variables and potential confounders.

## Results

Participants’ characteristics at baseline are shown in Table 2. We observed significant differences in some demographic aspects between the intervention and control group.

**Table 2:** Participants’ characteristics in the intervention and the control groups in the first phase of the study (Iran, 2017)

Characteristic	Intervention (***n***=133)	Control (***n***=133)	*P*-value
**Age (years)**	26.11± 4.99	31.55± 5.61	< 0.001
**Marital status**			
Married	40 (30.1)	99 (74.4)	< 0.001
Single	93 (69.9)	34 (25.6)	
**Education level**			
Middle school	9 (6.8)	9 (6.8)	0.520
High school	68 (51.1)	59 (44.3)	
Academic study	56 (42.1)	65 (48.9)	
**Employment status**			
Employed	100 (75.2)	125 (94.0)	< 0.001
Unemployed	33 (24.8)	8 (6.0)	

Values are presented as number (%) or mean± standard deviation.

A comparison of the frequency of tobacco consumption among the two groups in the two phases of the study is presented in Table 3. The frequency of hookah use and the duration of hookah consumption per serving in the second phase of the study compared to the first phase decreased significantly in the intervention group. There were no considerable changes in the control group. Although smoking cigarettes in both groups did not show any significant difference after the intervention. The end part of Table 3 represents a comparison of the changes in the frequency and duration of hookah consumption among the two intervention and control groups. As shown in this table, the frequency of hookah consumption decreased in 72.6% of participants in the intervention group (vs. 6.3% in the control group); and the duration of hookah consumption per serving decreased in 39.5% of participants in the intervention group (vs. 5.5% in the control group).

**Table 3:** Comparison of the frequency of tobacco consumption and its changes among two groups of the study (Iran, 2017)

Frequency of consumption	Intervention group		Control group	
	Phase 1	Phase 2	Phase1	Phase 2
**Frequency of hookah consumption**				
Once a week or less	24 (18.1)	64 (51.6)	30 (22.6)	28 (21.9)
More than once a week	15 (11.3)	47 (37.9)	76 (57.1)	62 (48.4)
Once per day	68 (51.1)	8 (6.5)	18 (13.5)	21 (16.4)
More than once per day	26 (19.5)	5 (4.0)	9 (6.8)	17 (13.3)
*P*-value	< 0.001		0.252	
**Duration of hookah consumption per serving**				
Less than 30 min	32 (24.1)	50 (40.3)	9 (6.8)	5 (3.9)
30–60 min	56 (42.1)	63 (50.8)	59 (44.4)	62 (48.4)
More than 60 min	45 (33.8)	11 (8.9)	65 (48.8)	61 (47.7)
*P*-value	< 0.001		0.536	
**Cigarette smoking status**				
Never smoker	73 (54.8)	76 (58.5)	73 (54.9)	64 (50.0)
Less than 10 cigarettes per week	34 (25.6)	19 (14.6)	19 (14.3)	24 (18.8)
10–20 cigarette per week	17 (12.8)	23 (17.7)	27 (20.3)	29 (22.7)
More than 20 cigarettes per week	9 (6.8)	12 (9.2)	14 (10.5)	11 (8.6)
*P*-value	0.133		0.680	
**Change in the frequency of hookah consumption**				
Unchanged or increased	34 (27.4)		120 (93.8)	
Decreased	90 (72.6)		8 (6.3)	
*P*-value	< 0.001			
**Change in the duration of hookah consumption per serving**				
Unchanged or increased	75 (60.5)		121 (94.5)	
Decreased	49 (39.5)		7 (5.5)	
*P*-value	< 0.001			

Values are presented as number (%).

We used two logistic models to evaluate the effect of the multidimensional intervention on the frequency and duration of hookah consumption by controlling for potential confounders (differences in the two groups at baseline: age, marital status, and employment status). The results indicated that being in the intervention group increased 72.1 (OR=72.1, 95% CI: 23.0–225.7, *P*< 0.001) and 24.1 (OR=24.1, 95% CI: 7.5–77.4, P< 0.001) times the odds of decreasing in the frequency of hookah consumption and decreasing in the duration of hookah consumption per serving, respectively.

Mean scores according to SEM levels are given in Table 4. After adjustment for the value of the dependent variable in phase 1, age, marital status, and employment status, at the individual level, the mean perceived sensitivity in the intervention group was higher at the end of the study compared to that of the control group. Moreover, the mean perceived severity in the intervention group was higher compared to its rate in the control group. As it is displayed in this table, after adjustment for the value of the dependent variable in phase 1, age, marital status, and employment status, at the social level, the mean perceived reward was lower and the perceived social support was higher among intervention group in comparison to participants in the control group.

**Table 4:** Personal, social, and environmental levels of SEM before and after the multilevel intervention between the two groups (Iran, 2017)

level of SEM	Phase 1		Phase 2		Adjusted^a^ mean for phase 2		*P*-value
	Intervention group	Control group	Intervention group	Control group	Intervention group	Control group	
**Perceived sensitivity**	11.7± 2.1	12.6± 2.4	16.2± 1.7	12.9± 2.1	16.5± 1.7	12.8± 2.2	< 0.001
**Perceived severity**	25.9± 3.5	25.2± 3.1	30.6± 2.5	25.5± 3.0	30.5± 2.6	25.6± 3.1	< 0.001
**Perceived rewards**	32.4± 5.6	35.1± 5.2	19.1± 5.2	34.5± 6.1	20.3± 5.1	33.8± 6.1	< 0.001
**Social support**	35.7± 6.4	36.7± 4.0	43.6± 4.4	35.6± 3.6	43.9± 4.5	35.3± 3.6	< 0.001

Values are presented as mean± standard deviation.

^a^Adjusted for the value of the dependent variable in phase 1, age, marital status, and employment status

Concerning contextual changes in coffee houses, it should be noted that in the first phase of the study, from among the 7 coffee houses existing in QarahAghaj city (the control group), only two of them served food before the intervention. Of the 11 coffee houses in the intervention group, 5 of them served light food and one had games like chess and mensch. One of the coffee houses in the intervention group was omitted from the study due to the owner’s avoidance to cooperate with the researchers during the study. Before the intervention, 9.1% of the coffee houses had games and entertainment facilities while such facilities were available in all the coffee houses after the intervention. At the beginning of the intervention, 83% of a total of 6 coffee house owners who provided customers with games and food were satisfied with the changes applied while after the intervention, 100% of the owners of 10 coffee houses were satisfied with the environmental changes. Besides, coffee house owners stated the changes had been effective in raising their income. Control group coffee houses did not show any changes in the level of owner satisfaction or income since environmental variables had remained intact.

## Discussion

The purpose of this study was to determine the effect of an intervention based on an SEM on reducing hookah use among the adolescents and adults in the city of Hashtroud. The results indicate that the intervention reduced hookah smoking significantly. Moreover, the frequency of hookah use and the duration of smoking hookah in one session showed a significant decrease in the intervention group but not in the control group. The frequency of hookah consumption decreased in 72.6% of participants in the intervention group (vs. 6.3% in the control group); and the duration of hookah consumption per serving decreased in 39.5% of participants in the intervention group (vs. 5.5% in the control group). Evidence from other studies supports our results. An intervention study conducted in Israel resulted in a reduction of hookah smoking of 22.2.% [10]. In the Lipkus et al. study [11], the effect of the intervention was a reduction of 62% which was almost similar to the result of the present study.

Most studies show, that individuals smoke hookah and cigarettes simultaneously [5]. According to the results of the present study at baseline, about 45% of hookah smokers in both groups were cigarette smokers as well. Thus, it can be stated, that interventions in tobacco use must be designed in a way that reduction in the use of one type of smoking would not lead to an increase in the use of another. Consistent with the previous studies [10, 25, 26] there was no significant change in cigarette smoking status between two phases of study in the intervention group.

The results of this study showed that after the intervention, individual-level factors in SEM (perceived severity and perceived sensitivity) in the intervention group significantly were higher than the control group by adjusting for their values in the baseline and potential confounders. Consistent with the present study, the study of Sadeghi et al. showed the same result between the intervention and control group [6]. Different studies showed that the higher the perceived sensitivity about a particular behavior, the more the individual considers him/herself exposed to illness and the more willingly takes action to create positive behavioral changes [27–29]. Moreover, the perceived intensity can make health problems caused by smoking seriously in the eyes of the individuals regarding their physical, psychological, and social effects [27]. Different studies show that perceived sensitivity and intensity are two of the most important predictors for conducting certain behavior or refraining from doing so [27, 29].

At the social level, in comparison to the control group, the adjusted mean of perceived reward in the intervention group considerably was low and the adjusted mean of social support in the intervention group considerably was high. Studies have indicated that the perceived reward gained from substance use including feelings of greatness, pride, etc. is one of the most important factors that predict such behavior [5, 29]. On the same basis, messages whose content was a positive perceived reward in order not to smoke hookahs replaced negative rewards through the telegram virtual network. It may be concluded, that using social networks for sending messages to individuals with positive reward content can have a significant effect on quitting smoking hookahs or reducing its use. Studies show that individuals smoke hookahs with their close friends [5] and behaviors are normally reinforced inside networks of friends. Moreover, cigarette and hookah users consider smoking an enjoyable social amusement which reinforces the feeling of togetherness among different individuals [30]. Interestingly, the decision to quit smoking is usually not made individually but such decisions are often made by a group of individuals connected directly or indirectly in a network [31].

According to the ecological approach to the creation of changes at individual and social levels, it is necessary to pave the way for environmental changes and establish such changes. This study showed that effective low-cost changes can be made in coffee houses very easily. We conducted the study in Hashtroud and QarahAghaj – small towns with fewer leisure opportunities than larger cities. Thus, coffee houses are accessible and cheap options to be used as places for friendly get-togethers. In coffee houses, drinking tea and smoking hookah is common. The intervention in this study was able to reduce hookah use in the coffee houses an interesting aspect of this study was that changes took place at all levels simultaneously. At the individual level, the perceived sensitivity and intensity was increased. At the social level, the virtual network entitled “saying no to hookahs” was formed. An increase in relations among friends to reduce hookah use, change in perceived rewards, and creation of social support through social networks, sending messages and discussions by lay persons and participants in virtual groups simultaneous with environmental changes in coffee houses all resulted in a kind of aggregation in efforts to reduce hookah use. As can be seen in different studies simultaneous changes at different levels can be effective in increasing health behavior [17]. All the owner’s coffee houses reported that the income from the sale of desserts and food was good for them. However, we did not investigated the income changes of the owners in the coffee houses before and after the intervention, which may be considered as a limitation for the study.

## Conclusion

The present study was able to reduce hookah use by the creation of environmental changes in coffee houses as well as through the establishment and development of social networks and making use of virtual networks’ capabilities to increase social support and perceived reward gained from quitting hookah use and sending messages to change the perceived sensitivity and intensity at the individual level. Therefore, it can be concluded that using multi-level strategy interventions would play a considerable role in reducing hookah use. The results of this study can be useful for policy-makers and consultants in the field of narcotics consumption control as well as researchers in this field.

## Supplementary Information

**Additional file 1.** Hookah Use Questionnaire. The validity and reliability of this researcher-made questionnaire have approved in this study.

**Additional file 2.** Social support for hookah smoking Questionnaire. This instrument was the researcher-made questionnaire that developed in this study. In the present study to investigate the factors at the social level were used two questionnaires including perceived reward of tobacco use that has been validated in a previous study (24) and the social support for hookah smoking questionnaire that was developed in this study.

**Additional file 3.** Environmental cheklist for coffe shop. To investigate the factors at the physical environmental level, environmental cheklist for coffe shop was developed and applied in the present study.

## Data Availability

The data collection tools and datasets generated and/or analyzed during the current study are available from the corresponding author on reasonable request.

## Abbreviations

SEM: Social-ecological model
EMRO: Eastern mediterranean region
CVR: Content validity ratio
CVI: Content validity index
