# Supplementary material for: Effects of a multi-level intervention on hookah smoking frequency and duration among Iranian adolescents and adults: an application of socio-ecological model
Source: BMC Public Health. 2021 Jan 21;21:184. doi: 10.1186/s12889-021-10219-8 (PMC7818729; doi:10.1186/s12889-021-10219-8)
Supplement: Supplementary file 1 — Additional file 1. Hookah Use Questionnaire. The validity and reliability of this researcher-made questionnaire have approved in this study. [file 12889_2021_10219_MOESM1_ESM.docx]

**Additional file 1**

**Title of data:** Hookah Use Questionnaire

In previous 3 months, how many times per week have you smoked hookah?

A) Once a week or less B) More than once a week

C) Once a day D) More than once a day

How long is the duration of your hookah smoking per serving?

A) Less than 30 minutes B) 30-60 minutes C) More than 60 minutes

How many cigarettes have you smoked in previous seven days?

A) Never smoker B) Less than 10 cigarette per week

C) 10-20 cigarette per week D) More than 20 cigarette per week
