# Supplementary material for: Effects of a multi-level intervention on hookah smoking frequency and duration among Iranian adolescents and adults: an application of socio-ecological model
Source: BMC Public Health. 2021 Jan 21;21:184. doi: 10.1186/s12889-021-10219-8 (PMC7818729; doi:10.1186/s12889-021-10219-8)
Supplement: Supplementary file 2 — Additional file 2. Social support for hookah smoking Questionnaire. This instrument was the researcher-made questionnaire that developed in this study. In the present study to investigate the factors at the social level were used two questionnaires including perceived reward of tobacco use that has been validated in a previous study (24) and the social support for hookah smoking questionnaire that was developed in this study. [file 12889_2021_10219_MOESM2_ESM.docx]

**Additional file 2**

**Title of data:** **Social support for hookah smoking questionnaire**

In the following 14 questions, please describe the support you have received from your friend(s) about using hookah during the previous 3 months.

.1- Smoked hookah with you.

Never 2)Seldom 3)Sometimes 4)Often 5)Always

2- Encouraged you not to go to the coffee shops to smoke hookah.

Never 2)Seldom 3)Sometimes 4)Often 5)Always

3- Encouraged you not to smoke hookah.

Never 2)Seldom 3)Sometimes 4)Often 5)Always

4- Changed their daily schedule so you could smoke hookah together.

Never 2)Seldom 3)Sometimes 4)Often 5)Always

5- Offered you to go to the coffee shop together.

Never 2)Seldom 3)Sometimes 4)Often 5)Always

6- Offered you to smoke hookah together.

Never 2)Seldom 3)Sometimes 4)Often 5)Always

7- Planned for some entertainments and recreational activities other than hookah smoking.

Never 2)Seldom 3)Sometimes 4)Often 5)Always

8- Talked to you about hookah smoking

Never 2)Seldom 3)Sometimes 4)Often 5)Always

9- Talked to you about decreasing of hookah smoking frequencies

Never 2)Seldom 3)Sometimes 4)Often 5)Always

10- Helped you in planning for activities aiming at decreasing hookah smoking frequencies.

Never 2)Seldom 3)Sometimes 4)Often 5)Always

11- Asked you for ideas on how they could get less hookah smoked.

Never 2)Seldom 3)Sometimes 4)Often 5)Always

12- Got angry at you for hookah smoking.

Never 2)Seldom 3)Sometimes 4)Often 5)Always

13- Criticized you or mucked you for decreasing hookah smoking frequencies.

Never 2)Seldom 3)Sometimes 4)Often 5)Always .

14- provided you with rewards after decreasing hookah smoking frequencies

Never 2)Seldom 3)Sometimes 4)Often 5)Always
