# Supplementary material for: Effects of a multi-level intervention on hookah smoking frequency and duration among Iranian adolescents and adults: an application of socio-ecological model
Source: BMC Public Health. 2021 Jan 21;21:184. doi: 10.1186/s12889-021-10219-8 (PMC7818729; doi:10.1186/s12889-021-10219-8)
Supplement: Supplementary file 3 — Additional file 3. Environmental cheklist for coffe shop. To investigate the factors at the physical environmental level, environmental cheklist for coffe shop was developed and applied in the present study. [file 12889_2021_10219_MOESM3_ESM.docx]

**Additional file 3**

**Title of data: Environmental cheklist for coffe shop**

1. Is there a suitable place to eat food and dessert in the coffee shop? Yes No
2. Are there any healthy light foods like lentil soup, omelet, etc. in the coffee shop? Yes No
3. Are there any healthy desserts like figs and broad beans , etc. in the coffee shop? Yes No
4. Is there any game/ entertainment instrument available in the coffee shop? Yes No
5. Is there a space in the coffee shop to play intellectual game accessories like chess and mensch, etc? Yes No
6. Do the customers use the coffee house game facilities? Yes No
7. Do the customers use the foods served in the coffee shop? Yes No
8. Is the coffee shop owner satisfied with changes such as serving food and playing games in the coffee shop? Yes No
9. Are the customers satisfied with the entertainment options available in the coffee shop? Yes No
10. In your idea, do the entertainment options available in the coffee shop have had effects on reducing hookah consumption in customers? Yes No
11. In your idea, do the entertainment options available in the coffee shop have resulted in increasing the income of the owner(s)? Yes No
